# Concise network models of memory dynamics reveal explainable patterns in path data

**DOI:** 10.1126/sciadv.adw4544

**Published:** 2025-10-10

**Authors:** Rohit Sahasrabuddhe, Renaud Lambiotte, Martin Rosvall

**Affiliations:** ^1^ Mathematical Institute, University of Oxford, Oxford OX2 6GG, UK.; ^2^ Institute for New Economic Thinking, University of Oxford, Oxford OX1 3UQ, UK.; ^3^ Integrated Science Lab, Department of Physics, Umeå University, Umeå 90736, Sweden.

## Abstract

Network methods capture the interplay between structure and dynamics of complex systems across scales by modeling indirect interactions as random walks. However, path data from real-world systems frequently exhibit memory effects that this first-order Markov model fails to capture. Although higher-order Markov models can capture these effects, they grow rapidly in size and require large amounts of data, making them prone to overfitting some parts and underfitting others in systems with uneven coverage. To address this challenge, we construct concise networks from path data by interpolating between first-order and second-order Markov models. We prioritize simplicity and interpretability by creating state nodes that capture prominent second-order effects and by proposing a transparent measure that balances model size and quality. Our concise networks reveal large-scale memory patterns in both synthetic and real-world systems while remaining far simpler than full second-order models.

## Teaser

Concise network models interpolating between Markov orders balance simplicity and quality of fit to reveal patterns in path data.

## INTRODUCTION

Networks model complex systems by abstracting their components as nodes and interactions as edges (*1*). These edges often represent flows of some quantity, such as passengers between airports, information between people, or workers between occupations. Although edges represent only direct flows, a defining feature of networks is their ability to capture indirect flows through paths or walks. This capacity allows researchers to analyze complex systems across scales (*2*), revealing mesoscale structures such as communities and roles and macroscale patterns such as hierarchies and rankings. Capturing these patterns assumes that flows are transitive: Given flow from node *i* to node *j* and from *j* to node *k*, there is implied indirect flow from *i* to *k* through *j*, often modeled as a first-order Markov process.

In a first-order network model of trajectory data, nodes represent system components and edges capture transition rates between them. However, sequence data from many real-world systems exhibit higher-order dependencies (*3*–*6*) that they fail to capture. To address this issue, researchers have developed network models with memory (*7*–*11*), extending the network toolbox to contexts where the Markovian assumption breaks down. A natural extension is a second-order network, which incorporates one-step memory using state nodes of the form *j*|*i*—indicating arrival at *j* from *i*, where *j* and *i* are nodes in the first-order network, first-order nodes for short. This approach generalizes to build networks with fixed-order memory of any order, enabling models that capture increasingly complex dependencies.

Modeling an entire system with a single Markov order imposes a strong constraint because real systems often exhibit dependencies spanning multiple orders. Combining Markov models up to a maximum order can improve next-element prediction in real-world systems (*10*, *12*). However, uneven observation density can cause such models to overfit some regions and underfit others. An alternative is to add state nodes only where needed (*9*, *13*). Although these approaches help mitigate overfitting, they rely on heuristics to estimate the importance of memory effects, limiting their interpretability and scalability even for moderately sized systems. Effective models must strike a balance between simplicity and predictive accuracy (*14*).

In this work, we build principled and interpretable models that interpolate between first-order and second-order networks. Using nonnegative matrix factorization (NMF) to identify prominent modes in second-order dynamics, we construct coarse-grained state nodes that represent these patterns instead of individual predecessors. To balance quality and complexity, we introduce a simple performance measure and incorporate an empirical Bayes-style regularization to mitigate overfitting. We validate our method on synthetic data and apply it to two real-world systems: air travel and information spread. In both cases, the method captures essential memory effects with a small number of interpretable state nodes, providing insights beyond those revealed by first-order models.

## RESULTS

### Concise network models of path data

**Fig. 1.**
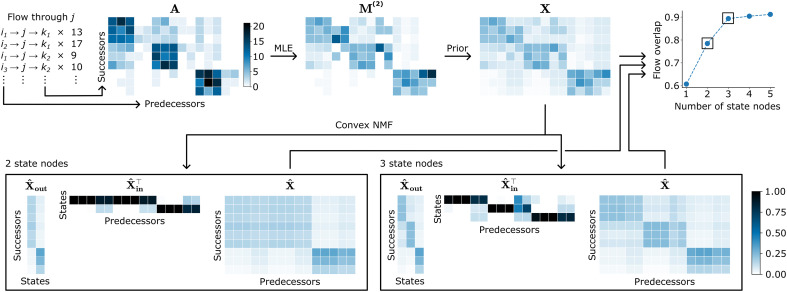
Method schematic. We illustrate our method using transitions through a first-order node (top left). (i) Input data: Matrix A counts observations, with rows showing successors and columns showing predecessors. This example shows three blocks; predecessors connect primarily to successors in the corresponding block, and the first and second blocks share more connections. Two predecessors in each block have sparse data. (ii) MLE: The MLE $M^{\left(2\right)}$ of the second-order transition probabilities overfits to the undersampled predecessors and creates unreliable transition probabilities. (iii) Regularization: Our prior smooths these extremes and creates target second-order dynamics in matrix X that balance observed patterns with statistical reliability. (iv) Pattern discovery: We create state nodes by decomposing X using convex NMF into transition matrices ${\hat{X}}_{\text{out}}$ and ${\hat{X}}_{\text{in}}$ , which capture transitions out of and into the state nodes. (v) Concise models: Models with two and three state nodes recover the planted patterns. Two state nodes merge the similar first and second blocks, whereas three state nodes distinguish them. In both cases, well-sampled predecessors transition mainly to single state nodes whereas undersampled predecessors spread across multiple state nodes. (vi) Quality assessment: Flow overlap measures how well our approximation $\hat{X}$ captures the target X . In this example, adding more than three state nodes achieves minimal improvement, indicating that three state nodes capture the essential large-scale patterns.

Understanding system dynamics requires models that balance simplicity with explanatory power. Our approach tackles three competing demands: capturing memory effects that first-order models miss, preventing the overfitting that plagues second-order models, and maintaining the interpretability that users need. We construct concise and interpretable network representations that capture key memory effects in path data, interpolating between first-order and second-order Markov models. To this end, we split first-order nodes into state nodes that capture prominent second-order effects. We illustrate the steps we follow for first-order node *j* with an example in Fig. 1. Consider data in the form of trajectories through a network, each of which is a sequence of nodes. We extract the flow through *j* into matrix A of observation counts such that $A_{\mathrm{ki}}$ is the frequency of the trigram i→j→k . We omit the dependence on *j* from our notation because the following steps are carried out separately for each *j* and use *i* and *k* to refer to predecessors and successors, respectively. Overlap in the sets of predecessors and successors has no effect on our method. Let matrix $M^{\left(2\right)}$ be the maximum likelihood estimate (MLE) of second-order transition rates, obtained by normalizing the columns of A.

$$M_{\mathrm{ki}}^{\left(2\right)}≔\frac{A_{\mathrm{ki}}}{\sum_{k^{\prime }}A_{k^{\prime }i}}$$ (1)

The MLE is susceptible to overfitting (see Fig. 1), which we mitigate by introducing a prior.

#### The prior

Let vector $M^{\left(1\right)}$ be the MLE of first-order transition rates from *j*

$$M_{k}^{\left(1\right)}≔\frac{\sum_{i^{\prime }}A_{ki^{\prime }}}{\sum_{k^{\prime },i^{\prime }}A_{k^{\prime }i^{\prime }}}$$ (2)

measuring the probability of moving from *j* to *k* independently of *i*. We apply a Dirichlet prior to each column $M_{\cdot i}^{\left(2\right)}$ with parameters $M^{\left(1\right)}$ and strength $\mu \in {\mathbb{R}}^{+}$ (see Methods). This corresponds to assigning a pseudocount of $\mu M_{k}^{\left(1\right)}$ to $A_{\mathrm{ki}}$ . The matrix X is the posterior mean of the second-order transition rates

$$X_{\mathrm{ki}}≔\frac{A_{\mathrm{ki}}+\mu M_{k}^{\left(1\right)}}{\sum_{k^{\prime }}A_{k^{\prime }i}+\mu }$$ (3)

We can write the column $X_{\cdot i}$ as

$$X_{\cdot i}=\left(\frac{\mu }{\mu +n_{i}}\right)M^{\left(1\right)}+\left(1-\frac{\mu }{\mu +n_{i}}\right)M_{\cdot i}^{\left(2\right)}$$ (4)

where $n_{i}=\sum_{k^{\prime }}A_{k^{\prime }i}$ . This interpolation balances memory and regularization: High μ pushes each column in X toward the memoryless model $M^{\left(1\right)}$ , whereas low μ preserves the patterns in $M^{\left(2\right)}$ . In cases where system knowledge cannot inform the strength of the prior μ, we use leave-one-out cross-validation (see Methods). The prior is stronger for undersampled predecessors, regularizing their transition rates (see Fig. 1). X contains the regularized dynamics through *j* that we aim to model with interpretable components.

#### Constructing state nodes

With reliable target dynamics established, we tackle the core challenge: discovering X’s hidden behavioral patterns that enable efficient representation. We approximate the target dynamics in X by splitting *j* into state nodes that capture prominent second-order patterns. Unlike full second-order state nodes that represent specific predecessor-successor pairs, our state nodes capture behavioral modes. For air traffic, for example, predecessor-successor pairs correspond to specific routes like arriving at Denver from Chicago, whereas our state nodes capture broader patterns such as eastbound transit or westbound transit. Each state node α has estimated transition probabilities from predecessors and to successors. $\hat{P}\left(i\to \alpha \right)$ is the probability that a random walker arriving at *j* from *i* uses α. $\hat{P}\left(\alpha \to k\right)$ is the probability that it moves to *k* from α.

$$X_{\mathrm{ki}}\approx {\hat{X}}_{\mathrm{ki}}≔\sum_{\alpha }\hat{P}\left(i\to \alpha \right)\hat{P}\left(\alpha \to k\right)$$ (5)

We can rewrite this expression as

$$\begin{array}{c} \;\hat{X}={\hat{X}}_{\text{out}}{\hat{X}}_{\text{in}}^{⊤},\text{where}\\ {\left({\hat{X}}_{\text{in}}\right)}_{i\alpha }≔\hat{P}\left(i\to \alpha \right)\\ {\left({\hat{X}}_{\text{out}}\right)}_{k\alpha }≔\hat{P}\left(\alpha \to k\right) \end{array}$$ (6)

For a given number of state nodes r≪min(∣i∣,∣k∣) , we use convex NMF to find the optimal approximation such that ${\hat{X}}_{\text{in}}$ and ${\hat{X}}_{\text{out}}$ are transition matrices (see Methods). We have omitted the dependence of the estimated matrices on *r* to simplify notation. Crucially, each column of ${\hat{X}}_{\text{out}}$ is a convex combination of the columns of X . This ensures that the state nodes model plausible behavior (see Fig. 1).

#### Model quality

For each first-order node, the trade-off between model size and description quality is made in the choice of *r*. This choice connects directly to our regularization: When μ preserves complex patterns, we need high *r* to capture them; when μ creates uniform behavior, low *r* suffices. We define a simple and intuitive measure of quality of fit to guide this choice in situations where it cannot be made with system knowledge. We define the flow overlap of two discrete probability distributions over the same domain as the probability mass in the same elements

$$\text{flow overlap}\left(p,q\right)≔\sum_{i}\text{min}\left[p\left(i\right),q\left(i\right)\right]$$ (7)

where *p* and *q* are discrete probability distributions over a set indexed by *i*. We can extend flow overlap to transition matrices as the (weighted) mean flow overlap of every column. For the rank *r* solution $\hat{X}$ , flow overlap becomes

$$\begin{array}{c} \text{flow overlap}\left(X,\hat{X}|n_{i}\right)≔\frac{1}{\sum_{i^{\prime }}n_{i^{\prime }}}\sum_{k,i}n_{i}\text{min}\left(X_{\mathrm{ki}},{\hat{X}}_{\mathrm{ki}}\right) \end{array}$$ (8)

This quantity ranges from 0 to 1 and measures the fraction of flow through the first-order node that our approximation $\hat{X}$ captures. When the target dynamics of all the predecessors are similar, possibly caused by a strong prior, the *r* = 1 solution achieves high flow overlap. At the other extreme, when each predecessor has very different behavior, flow overlap will remain low until *r* approaches min(∣i∣,∣k∣) . Our method works best when the target dynamics contain large-scale patterns, as in our example, where flow overlap increases rapidly until rank 3, after which adding state nodes captures no important new behavior (Fig. 1). We pick the optimal number of state nodes as the lowest *r* such that the flow overlap reaches a threshold.

#### Constructing the network

We can create state nodes independently in parallel for each first-order node. In large systems, we can also restrict the creation of state nodes to a subset of important first-order nodes. We put the concise network model together by linking state node $\alpha^{i}$ to $\beta^{j}$ with weight

$$\begin{array}{l} \hat{P}\left(\alpha^{i}\to \beta^{j}\right)={\left({\hat{X}}_{\text{out}}^{i}\right)}_{j\alpha^{i}}{\left({\hat{X}}_{\text{in}}^{j}\right)}_{i\beta^{j}}\\ \;=\hat{P}\left(\alpha^{i}\to j\right)\hat{P}\left(i\to \beta^{j}\right) \end{array}$$ (9)

where the superscript indicates the first-order node. Convex NMF tends to create sparse factors (*15*). Many of the entries of ${\hat{X}}_{\text{in}}$ and ${\hat{X}}_{\text{out}}$ will be close to 0, meaning that predecessors and successors interact primarily with a subset of state nodes. However, because the NMF is likely to converge before the weights are exactly 0, state nodes have dense neighborhoods with many low-weight edges. We recommend trimming these low-importance edges, for which we implement a simple threshold-based approach (see Methods).

### Synthetic experiments

We examine the performance of our method on synthetic first-order nodes with 50 predecessors and successors each. Defining *n_m_ modes*—distributions over the successors—we sample data for each predecessor from a convex combination of modes with weights drawn from a symmetric distribution with spread controlled by *c* > 0. The distribution is uniform for *c* = 1, and higher values decrease the variance. Intuitively, for c≪1 , each predecessor closely resembles a single mode and is a uniform mixture of them for c≫1 . We vary $n_{m}\in \left\{\;2,5,10\;\right\}$ and c∈{0.5,1.0,1.5} and generate 25 synthetic first-order nodes for each pair (see Methods for details).

**Fig. 2.**
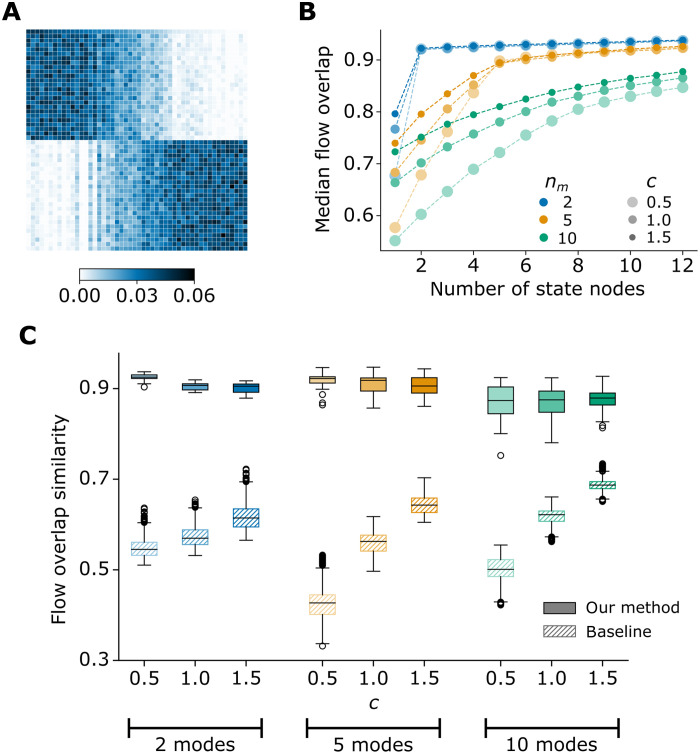
Synthetic experiments. (**A**) Typical example of X for *n_m_* = 2 modes and concentration parameter *c* = 0.5. Most predecessors closely resemble a single mode. (**B**) Median flow overlap as a function of the number of state nodes. We note that the single-state node solution corresponds to a first-order model. (**C**) Flow overlap similarity between the state nodes created by our method (solid) and the baseline (hatched) with the predecessors closest to the planted modes. We distinguish *n_m_* with colors and *c* with transparency.

First, we assess whether creating state nodes improves the quality of fit. For *n_m_* = 2,5, flow overlap increases rapidly with the addition of state nodes, plateauing at a high value after *n_m_* state nodes (Fig. 2B). Although this elbow-like behavior is less clear for *n_m_* = 10, flow overlap reaches high values of more than 0.8 with 10 state nodes. For the same *n_m_*, solutions with fewer than *n_m_* state nodes do better for higher *c*. This is expected because predecessors tend to be similar to each other for high values of *c*.

Second, we explore the interpretability of the model with *n_m_* state nodes. Because the planted modes are extreme behaviors unlikely to be observed in the data, we do not want the state nodes to match them exactly. Instead, we compare the state nodes to the predecessors, which are most similar to the modes (see Methods). We report the mean flow overlap similarity (Eq. 7) of the best matching of state nodes with these predecessors (Fig. 2C). As a baseline, we randomly generate 50 representations with *n_m_* state nodes for each first-order node (see Methods). Not only do we outperform the baseline for all values of the parameters, but we also achieve objectively high values of similarity (Fig. 2C).

### Transit flow through airports

To validate our method on real-world systems, we start with air traffic because airports handle millions of passengers whose itineraries create clear memory effects. Although second-order memory is important in modeling the flow of passengers through airports (*8*, *16*, *17*), full second-order networks can be impractically large and are prone to overfitting. Here, we investigate whether our concise network model can capture key memory effects with interpretable state nodes and offer insights beyond a first-order network. We use open-source data from the US Bureau of Transport Statistics to construct a dataset of around 4.3 million domestic transits through 435 airports in the United States. We are interested specifically in the role of airports as transit hubs and only consider nonreturn transits of the form i→j→k where i≠k (see Methods).

**Fig. 3.**
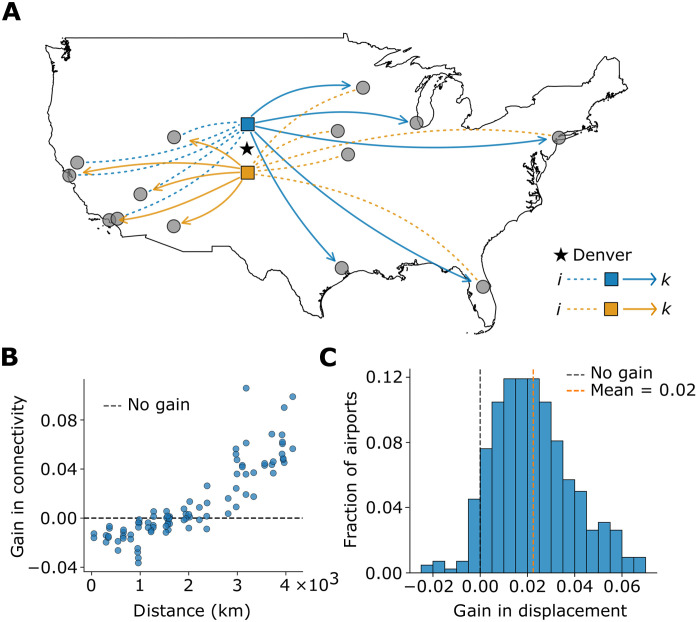
State nodes and connectivity in the airport network. (**A**) Denver with two state nodes. For either state node, we plot with gray circles the five predecessors [respectively, (resp.) successors] with the highest ${\left({\hat{X}}_{\text{in}}\right)}_{\mathrm{i\alpha}}$ $\left[\text{resp.}\;{\left({\hat{X}}_{\text{out}}\right)}_{\mathrm{k\alpha}}\right]$  from among the 20 most-observed predecessors (resp. successors) of Denver. The state nodes are denoted as blue and orange squares with edges of the same color. Dashed (resp. solid) lines are edges to (resp. from) state nodes. The black star marks the location of Denver. (**B**) Gain in three-leg connectivity (*y* axis)—${\text{log}}_{10}\left(\rho_{c}/\rho_{\text{fo}}\right)$—against the distance between *o* and *d* (*x* axis) for (o,d) pairs of airports ranked 11 to 20 by the number of transits. The black dashed line denotes no gain. (**C**) Distribution of gain in three-leg displacement—${\text{log}}_{10}\left(\delta_{c}/\delta_{\text{fo}}\right)$—for all origin airports except the 10 largest hubs. The black dashed line denotes no gain. The orange dashed line marks the mean.

The five largest airports by transit volume—in Atlanta, Dallas–Fort Worth, Denver, Charlotte, and Chicago—account for 42% of all transits, making it crucial to capture potential memory effects in their traffic. However, they have an average of 170 connections each, and a full second-order model is impractical. We explore whether we can build a more concise model by identifying meaningful modes of behavior (see note S2.1). Flow overlap increases rapidly between rank 1 and rank 2 or 3, indicating large-scale patterns that can be modeled with only a few state nodes. For instance, two state nodes for Denver increases flow overlap from 0.60 to 0.74 by capturing intuitive behavior—passengers arriving from the east are likely to continue westward and vice versa (Fig. 3A). We observe similar patterns for the other large national hubs, revealing that passengers use them to travel between distant regions.

First-order networks cannot model this behavior, and modeling memory effects changes connectivity analysis in transport systems. We analyze this by constructing (i) a first-order network $G_{\text{fo}}$ and (ii) a concise memory network $G_{c}$ with state nodes for the 10 largest airports, which account for 57% of the transits (see Methods). Using a flow overlap threshold of 0.7, $G_{c}$ has 27 state nodes for these airports and is much smaller than a network with full second-order models for them, which would have 1467 state nodes.

Modeling a passenger’s itinerary as a random walk on the network, we define three-leg connectivity $\rho_{\text{fo}}\left(o,d\right)$ [respectively, (resp.) $\rho_{c}\left(o,d\right)$ ] as the probability that a passenger starting at origin *o* reaches destination *d* in 3 or fewer steps on the first-order (resp. concise) network (see Methods). Comparing $G_{c}$ to $G_{\text{fo}}$ , the gain in connectivity is ${\text{log}}_{10}\left(\rho_{c}/\rho_{\text{fo}}\right)$ . For (o,d) pairs in the next 10 largest airports, the gain (i) increases with the geographic distance between *o* and *d* (Pearson *r* = 0.88, Kendall τ = 0.74, both with *P* value < 10^−16^) and (ii) is positive for all pairs of airports more than 2500 km apart (Fig. 3B). This result shows that first-order models systematically underestimate connectivity between distant airports, missing how passengers navigate the network through major hubs to traverse continental distances efficiently (fig. S12).

Covering larger distances on $G_{c}$ is not unique to journeys from big airports. Let $\delta_{\text{fo}}\left(o\right)$ [resp. $\delta_{c}\left(o\right)$ ] be the expected displacement from origin *o* after three steps on $G_{\text{fo}}$ (resp. $G_{c}$ ). The gain in three-leg displacement—${\text{log}}_{10}\left(\delta_{c}/\delta_{\text{fo}}\right)$—is positive for most origin airports (Fig. 3C). The mean gain = 0.02 is significantly greater than 0 (one-tailed *t* test statistic = 27.2, *P* value < 10^−16^). By capturing the role of large national hubs in routing traffic across distant regions, $G_{c}$ shows that passengers can travel long distances in a few flights.

### Group structure in information flow

Information flow is an important process in the analysis of social networks, where individuals are modeled as nodes and their interactions as edges. In reality, people interact in many different contexts, and whom you pass information on to likely depends on whom you got it from. Using social networks of co-work and friendship among 71 lawyers at a firm (*18*), we generate synthetic trajectories where information received from a friend (resp. co-worker) is passed on to a friend (resp. co-worker). From these, we construct (i) the first-order network $G_{\text{fo}}$ , (ii) a concise network $G_{c}$ with flow overlap threshold = 0.9, having two state nodes each for 52 individuals, and (iii) the second-order network $G_{\text{so}}$ (see Methods).

**Fig. 4.**
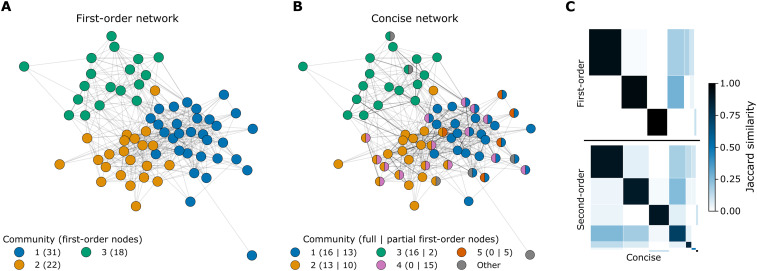
Group structure of information flow in the social network. Communities identified by Infomap in the (**A**) first-order and (**B**) concise networks. The circles are first-order nodes with colors indicating community membership. The legend includes community sizes, with the two smallest communities with fewer than five nodes each in $G_{c}$ labeled Other. Node positions are determined by the spring layout applied to $G_{\text{fo}}$ . (**C**) Jaccard similarity of first-order nodes in each community of $G_{c}$ (*x* axis) with those of $G_{\text{fo}}$ (*y* axis; top) and $G_{\text{so}}$ (*y* axis; bottom). The cells are ordered by community label, and their width (resp. height) is proportional to the size of the community in $G_{c}$ (resp. $G_{\text{fo}}$ and $G_{\text{so}}$).

Identifying group structures is key to understanding information spread. We use Infomap (*19*), a flow-based community detection tool, to find groups of people within which information circulates rapidly before spreading to the rest of the network (see Methods). The three communities of $G_{\text{fo}}$ (Fig. 4A) correlate with work-related metadata. Office location splits the individuals in community 3 from the rest, who are then divided into litigators and corporate lawyers (table S4).

For higher-order networks, Infomap works at a state node level, allowing communities to overlap at first-order nodes (*20*). $G_{c}$ has seven communities (Fig. 4B), of which two are very small. Communities 1 to 3 are nonoverlapping and are the same groups of co-workers identified in $G_{\text{fo}}$ (Fig. 4C, top). Communities 4 and 5 overlap with 1 and 2 and are novel to $G_{c}$ . They are friendship groups of people who work in the same office and correspond exactly to communities in the friendship network (note S3.3). This analysis shows that $G_{c}$ effectively captures overlapping social circles, revealing distinct yet interconnected groups of friends and co-workers. Despite having more than seven times as many nodes, the community structure of $G_{\text{so}}$ is very similar to that of $G_{c}$ (Fig. 4C, bottom), showing that we can model the important memory effects with far fewer nodes.

## DISCUSSION

We introduce a method to construct concise network models from path data. Using NMF, we create interpretable state nodes that reveal large-scale patterns in second-order dynamics. To prevent overfitting, we incorporate an empirical Bayes-style regularization and define a simple performance measure that balances model size and quality. Our approach produces compact networks that retain essential memory effects in empirical data at a fraction of the complexity of full second-order models.

Although we prioritize simplicity and interpretability, our framework is modular. Each stage of the pipeline can be adjusted to suit the system under study—for instance, by using cross-validation or statistical model selection criteria to choose the number of state nodes, applying more sophisticated methods for edge pruning, or experimenting with alternative regularizers and loss functions in the matrix factorization step. These extensions offer promising directions for future work.

The flexibility and scalability of our method make it well suited for analyzing path data across diverse domains. A natural extension is to incorporate higher-order memory effects. Similar techniques could also help identify and model nonstationary patterns in paths or build compact representations of temporal networks.

## METHODS

### Constructing concise networks

#### The prior

We prevent overfitting the second-order transition probabilities by imposing a prior on the MLE. Each column of $M^{\left(2\right)}$ is a distribution over the successors, which can be viewed as the event probability parameters of a multinomial distribution. For the prior, we choose its conjugate prior, the Dirichlet distribution. This ensures that the posterior is a multinomial distribution, allowing us to assemble the columns of X.

We choose the MLE of the first-order transition rates $M^{\left(1\right)}$ as the parameters of the Dirichlet prior. This ensures that, when the prior is strong, the target dynamics resemble the memoryless model. Here, we assume that $M^{\left(1\right)}$ is a good estimate of first-order transition probabilities. This assumption is not essential to our framework. For instance, in cases where data are very sparse, a uniform prior or one conserving node degree might be more suitable. However, data that are insufficient to estimate a first-order model are unlikely to be usable for higher-order modeling.

##### Leave-one-out cross-validation to pick μ

The choice of the prior strength is critical as high values of μ wash out memory effects whereas low values fail to remove the noise from $M^{\left(2\right)}$ . In cases where system knowledge cannot inform the choice, we use leave-one-out cross-validation (*21*). From Eq. 3, the likelihood of observing i→j→k in a model trained on all other transitions is

$$\frac{A_{\mathrm{ki}}-1+\mu M_{k}^{\left(1\right)}}{n_{i}-1+\mu }$$ (10)

We pick the μ that maximizes the log-likelihood of every observed transition in a model trained on all the others, i.e.

$$\mu^{*}=\text{arg}\underset{\mu }{\text{max}}\sum_{k,i}A_{\mathrm{ki}}\text{log}\left[\frac{A_{ki}-1+\mu M_{k}^{\left(1\right)}}{n_{i}-1+\mu }\right]$$ (11)

Our choice of leave-one-out cross-validation is motivated by its closed-form objective function and ease of optimization. Using other methods such as *k*-fold cross-validation to estimate the parameter(s) of the prior does not affect the rest of our method.

#### Convex NMF to construct state nodes

NMF is a tool to create low-rank approximations of matrices with interpretable factors. The goal of NMF is to obtain factorizations of the form $X\approx \hat{X}={\text{FG}}^{⊤}$ , where F,G≥0 . Convex NMF constrains the space of solutions by requiring the columns of F to lie in the column space of X . Thus, we search for a factorization $\hat{X}={\text{XWG}}^{⊤}$ , where $W,G^{⊤}\geq 0$ . We use the multiplicative updates from (*15*) to optimize the loss function

$${\left\|\hat{X}-X\right\|}^{2}=\sum_{k,i}{\left({\hat{X}}_{\mathrm{ki}}-X_{\mathrm{ki}}\right)}^{2}$$ (12)

In each iteration, we update

$$G\leftarrow G⊙\sqrt{\left(X^{⊤}\text{XW}\right)⊘\left({\text{GW}}^{⊤}X^{⊤}\text{XW}\right)}$$

$$W\leftarrow W⊙\sqrt{\left(X^{⊤}\text{XG}\right)⊘\left(X^{⊤}{\text{XWG}}^{⊤}G\right)}$$

where ⊙ and ⊘ are element-wise multiplication and division, respectively, until the solution converges to the local optimum [see ref. (*15*) for proofs of correctness and convergence]. The solution is not unique. For instance, we can write ${\text{XWG}}^{⊤}=X\left({\text{WA}}^{-1}\right){\left({\text{GA}}^{⊤}\right)}^{⊤}$ for alternative solutions with the same loss. Requiring that the matrix factors are transition matrices eliminates this degeneracy. We set

$${\hat{X}}_{\text{out}}={\text{XWD}}_{W}^{-1}$$ (13)

$${\hat{X}}_{\text{in}}={\text{GD}}_{W}$$ (14)

where $D_{W}$ is the diagonal matrix of the column sums of W . The columns of ${\hat{X}}_{\text{out}}$ are convex combinations of the columns of X , making ${\hat{X}}_{\text{out}}$ column stochastic. The row sums of ${\hat{X}}_{\text{in}}$ will be close to 1, and we normalize them. For each *r*, we pick the solution with the lowest loss among several candidates with different initializations.

##### Initialization

We use the equivalence of convex NMF to soft *k*-means clustering (note S1) to pick good initial values. We initialize G to a smoothed version of the *k*-means clustering of the columns of X into *r* clusters

$$G_{\mathrm{i\alpha}}=\left\{\begin{array}{cc} 1, & \text{if column}\;i\in \text{cluster}\;\alpha \\ 0.2, & \text{otherwise} \end{array}\right.$$ (15)

We initialize W to a row-normalized version of G.

##### Convergence

The loss is guaranteed to be nonincreasing under the multiplicative updates (*15*). In the analyses in this work, we declare convergence when the relative decrease in loss over 10 iterations is less than 10^−4^.

#### Trimming the neighborhood of state nodes

We implement a simple method to trim out low-importance edges from the neighborhood of state nodes, improving the interpretability and sparsity of the network. For a first-order node *j* with rank *r*, the importance of state node α to predecessor *i* is ${\left({\hat{X}}_{\text{in}}\right)}_{\mathrm{i\alpha}}$—the probability that a trajectory from *i* passes through α. We retain edge i→α if

$${\left({\hat{X}}_{\text{in}}\right)}_{\mathrm{i\alpha}}\geq \frac{1}{r}\times \sigma$$ (16)

where σ is a parameter that controls the strictness of the trimming such that smaller values retain more edges. Similarly, we keep α→k if

$$\frac{{\left({\hat{X}}_{\text{out}}\right)}_{\mathrm{k\alpha}}}{\sum_{\beta }{\left({\hat{X}}_{\text{out}}\right)}_{\mathrm{k\beta}}}\geq \frac{1}{r}\times \sigma$$ (17)

### Synthetic examples

#### Synthetic data

We generate synthetic first-order nodes with 50 predecessors and successors each. For each predecessor, we sample 1000 observations from its “true” out-distribution over the successors, which is a random combination of *n_m_* modes. These planted modes are uniform distributions over disjoint equally sized subsets of the successors. For instance, when *n_m_* = 2, the two modes are uniform distributions over 25 successors each. A predecessor’s true distribution is a convex combination of modes with weights drawn from a symmetric Dirichlet distribution with concentration parameter *c*. Increasing *c* makes predecessors more similar on average. We vary $n_{m}\in \left\{\;2,5,10\;\right\}$ and c∈{0.5,1.0,1.5} , generating 25 first-order nodes for each combination.

#### Interpretability of state nodes

Particularly for higher values of *c*, predecessors are unlikely to closely resemble single modes. Therefore, the planted modes are not realistic behavior given the data and we do not want state nodes to match them. Instead, we evaluate the interpretability of state nodes by comparing them to the “extreme” predecessors that are most similar to the modes. For each mode, we identify the closest predecessor by finding the column of X that has the highest flow overlap with it. We report the mean flow overlap of the best matching of the state nodes and this set of predecessors.

##### The baseline

Each state node in our model is a convex combination of the columns of X with weights discovered by the NMF. For a baseline, we create the same number of state nodes with weights distributed uniformly at random. We generate 50 instances of the baseline for each synthetic first-order node.

We have included the code to replicate this experiment in our GitHub repository (https://github.com/rohit-sahasrabuddhe/concise-networks) and on Zenodo (https://doi.org/10.5281/zenodo.15599985).

### Transit flow through airports

#### Data description

Our dataset is a 10% sample of domestic flight itineraries in the United States in the first quarter of 2023 (*22*). We discard all itineraries with just one leg and parse the rest into transits through each airport. Because we are interested in the role of airports as transit hubs, we remove return transits of the form i→j→i . This leaves 4,340,809 transits between 435 airports. Of these, 379 have transits through them, whereas the rest only serve as sources or destinations. We plot the distribution of transit volume (fig. S1) and the location of the airports (fig. S2) in the Supplementary Materials.

#### Constructing the networks

We create the first-order network $G_{\text{fo}}$ by setting rank = 1 for each first-order node. For the concise memory network $G_{c}$ , we create state nodes for the 10 largest transit hubs (table S1) with flow overlap threshold 0.7. $G_{c}$ has two state nodes each for Atlanta, Dallas–Fort Worth, Denver, Charlotte, Chicago, Seattle, and Houston, three for Minneapolis–St Paul, and five each for Phoenix and Las Vegas.

##### Backboning

We remove low-importance edges in two stages. First, we trim the neighborhoods of the state nodes in $G_{c}$ with σ = 0.05. Next, we use the disparity filter (*23*) to backbone both networks with a disparity filter score threshold of 0.01 (see note S2.2). We ensure that both networks remain weakly connected. After backboning, $G_{\text{fo}}$ has 435 nodes and 9249 edges and $G_{c}$ has 452 nodes and 12,429 edges.

#### Connectivity analysis

We model an itinerary as a discrete time random walk on the network. Let $T_{\text{fo}}$ be the row-stochastic adjacency matrix of $G_{\text{fo}}$ , where we give the three airports with no out-edges self-loops with weight = 1.

##### Three-leg connectivity

The probability that a passenger at origin *o* reaches destination *d* in three or fewer steps on $G_{\text{fo}}$ is given by

$$\rho_{\text{fo}}\left(o,d\right)≔{\left[{\left(T_{\text{fo}}^{d}\right)}^{3}\right]}_{\mathrm{od}}$$ (18)

where $T_{\text{fo}}^{d}$ is $T_{\text{fo}}$ modified to make *d* an absorbing state. Specifically, ${\left(T_{\text{fo}}^{d}\right)}_{\mathrm{id}}=0$ ∀i≠d and ${\left(T_{\text{fo}}^{d}\right)}_{\mathrm{dd}}=1$ . We define $\rho_{c}\left(o,d\right)$ similarly for cases where *o* and *d* have only one state each. In Results, we investigate the gain in three-leg connectivity between (o,d) pairs from the airports ranked 11 to 20 by transit volume (table S2).

##### Three-leg displacement

The expected three-leg displacement from *o* for $G_{\text{fo}}$ is

$$\delta_{\text{fo}}\left(o\right)≔\sum_{d}{\left[{\left(T_{\text{fo}}\right)}^{3}\right]}_{\mathrm{od}}\times \text{distance}\;\left(o,d\right)$$ (19)

We define $\delta_{c}$ similarly. In Results, we investigate the gain in three-leg displacement for all origins *o* except the 10 largest airports. In both analyses, we use Haversine distance to quantify geographic separation.

### Group structure in information flow

#### Data description

The Lazega law firm data (*18*) contain social networks of three relationships—co-work, friendship, and advice—between 71 lawyers at a corporate law firm. We use metadata on the practice (litigation or corporate) and office location (Boston, Hartford, or Providence) of the individuals (table S3).

#### Synthetic trajectories

We start with two separate social networks $G_{w}$ of co-work and $G_{f}$ of friendship. For simplicity, we make them undirected by discarding nonreciprocated edges. $G_{w}$ and $G_{f}$ contain 378 and 176 edges, respectively, of which 74 are shared. We generate trigrams i→j→k through every node *j* using a second-order Markov process to model information spreading separately along co-work and friendship links. If *i* is only a co-worker (resp. friend) of *j*, *k* is chosen uniformly at random from the set of co-workers (resp. friends). If *i* is both, *k* is picked from the disjoint union of friends and co-workers. For each *j*, we generate 1000 trigrams from each *i*.

#### Constructing the networks

We create $G_{\text{fo}}$ by setting rank = 1 for each first-order node. For $G_{c}$ , we pick a flow overlap threshold of 0.9 and create two state nodes each for 52 nodes. The full second-order network $G_{\text{so}}$ has ∣i∣ state nodes for each first-order node, with ${\hat{X}}_{\text{out}}=X$ and ${\hat{X}}_{\text{out}}=I$ , the identity matrix. We trim the neighborhoods of the state nodes in both higher-order networks with σ = 0.05. $G_{\text{fo}}$ has 71 nodes and 960 edges, $G_{c}$ has 123 nodes and 1293 edges, and $G_{\text{so}}$ has 960 nodes and 12,384 edges.

#### Community structure

Community detection is a long-studied task in network science with a plethora of approaches (*24*). We use Infomap (*19*), a method designed for networks of flow, and set its Markov time parameter to 0.9 (see note S3.1).

Infomap works at a state node level, allowing first-order nodes to belong to multiple communities. Viewing a community as a set of first-order nodes, we compare communities in different networks (Fig. 4C) using Jaccard similarity. For sets *A* and *B*

$$\text{Jaccard similarity}\;\left(A,B\right)≔\frac{|A\cap B|}{|A\cup B|}$$ (20)

## Supplementary Materials

### This PDF file includes:

Supplementary Notes S1 to S3 Figs. S1 to S14 Tables S1 to S4 References
