# Supplementary material for: Concise network models of memory dynamics reveal explainable patterns in path data
Source: Sci Adv. 2025 Oct 10;11(41):eadw4544. doi: 10.1126/sciadv.adw4544 (PMC12513435; doi:10.1126/sciadv.adw4544)
Supplement: Supplementary file 1 — Supplementary Notes S1 to S3 Figs. S1 to S14 Tables S1 to S4 References [file sciadv.adw4544_sm.pdf]

Science Advances
AAAS

Supplementary Materials for

Concise network models of memory dynamics reveal explainable patterns in path data

Rohit Sahasrabuddhe *et al*.

Corresponding author: Rohit Sahasrabuddhe, rohit.sahasrabuddhe@maths.ox.ac.uk;
Renaud Lambiotte, renaud.lambiotte@maths.ox.ac.uk

Sci. Adv. **11**, eadw4544 (2025)
DOI: 10.1126/sciadv.adw4544

This PDF file includes:

Supplementary Notes S1 to S3
Figs. S1 to S14
Tables S1 to S4
References

Supplementary Note S1   Convex NMF and soft k-means clustering

K-means clustering can be written as a matrix factorization problem, where we want to approximate the data matrix $\mathbf{X}$ as

$$\mathbf{X} \approx \hat{\mathbf{X}} = \mathbf{F}\mathbf{G}^\top, \tag{S1}$$

where $\mathbf{F}$ has the cluster centroids and $\mathbf{G}$ the memberships. Convex NMF is analogous to a soft k-means clustering, with cluster centroids in the convex hull of the data and fuzzy cluster membership. Ref. (*31*) has a general discussion of the relationship between clustering and NMF.

Supplementary Note S2   Flight transits network

Supplementary Note S2.1   State nodes of the 5 largest hubs

We create models for the five largest airports with 1 - 10 state nodes. We see (1) a steep increase in flow overlap between ranks 1 and 2, and (2) quite high values of flow overlap by rank 3 (Fig. S3), indicating that important memory effects can be captured by a few large-scale patterns. To illustrate these patterns, we visualize solutions with manually picked number of state nodes – rank 2 for Dallas, Denver, and Chicago and rank 3 for Atlanta and Charlotte (Figs S4 - S8).

Each row depicts the locations of a state node's predecessors (left) and successors (right). Its marker size indicates the flow from (resp. to) airport to (resp. from) the hub. The color of i indicates $(\hat{X}_{\text{in}})_{i\alpha}$. The color of k shows how over-represented it is in the out-distribution of α compared to the other state nodes:

$$(\hat{X}_{\text{out}})_{k\alpha} - \frac{\sum_{\alpha'} (\hat{X}_{\text{out}})_{k\alpha'}}{r}, \tag{S2}$$

where r is the number of state nodes.

The maps show clear geographic patterns that capture intuitive behavior – passengers use large national hubs to travel between distant regions. Travelers staying within a region are probably served by smaller regional hubs or direct flights.

Supplementary Note S2.2   Constructing the networks

We construct G_{fo} with 1 state node for each airport, and G_{c} with state nodes for the 10 largest airports (flow overlap threshold = 0.7, TableS1). Peaks near 1 in the distributions of the relative

strength of the prior (Fig. S9) show that $\mathbf{M}^{(2)}$ overfits to some predecessors.

Trimming edges At this stage, G_{fo} has 435 nodes and 10,222 edges, and G_{c} has 452 nodes and 15,233 edges. We first trim the neighborhoods of the state nodes in G_{c} using $\sigma = 0.05$, which removes 1,309 edges. Next, we apply the Disparity Filter (*23*) (implementation by Michele Coscia (*30*)) to remove low-importance edges from both networks.

Backboning The DF assigns a score to an edge by comparing its weight to a degree-preserving null model that randomizes the weight distribution of the edges of a node. We see a peak near 0 in the distribution of DF score for both networks S10. Let us define the *flow* along edge $i \to j$ as $f_{ij} = \text{PageRank}(i) \times w_{ij}$, where w_{ij} is the weight. This can be viewed as the amount of probability mass moving along an edge at stationary state. Since the networks are not strongly connected, we proxy the stationary state distribution with PageRank ($\alpha = 0.85$). Thus, $\sum_{i,j} f_{ij} = 1$. In Fig. S11, we plot the fraction of edges and flow retained for DF score threshold $\in [10^{-4}, 10^{-1}]$. With a threshold of 0.01, G_{fo} has 435 nodes and 9,249 edges, and G_{c} has 452 nodes and 12,429 edges. We ensure that both networks remain weakly connected.

Supplementary Note S2.3 Connectivity analysis

Generalizing the notation in the main text, we define $\rho_{\text{fo}}(o, d, t)$ as the t-leg connectivity from o to d on G_{fo}.

$$\rho_{\text{fo}}(o, d, t) := \left(\left(\mathbf{T}_{\text{fo}}^{\mathbf{d}}\right)^{t}\right)_{od}, \tag{S3}$$

where $\mathbf{T}_{\text{fo}}^{\mathbf{d}}$ is $\mathbf{T}_{\text{fo}}$ modified to make d an absorbing state. Fig. S12 shows that the analysis in the main text is robust to t.

Supplementary Note S3 Information flow network

Supplementary Note S3.1 Community detection

The Map Equation (*32*) is a function that maps a partition of nodes to the description length of a random walk on the network using a coding scheme that leverages group structure. It takes low values for partitions where the the random walker tends to remain within modules before spreading

to the rest of the network. Infomap (*19*) is a greedy algorithm to optimize the Map Equation. Infomap works at the level of state nodes, allowing (1) first-order nodes to be in multiple communities and (2) communities to overlap.

Supplementary Note S3.2 Picking Markov time

The Markov time parameter can be used to find group structure across scales (*33*) – with lower values creating finer modules. To pick a scale to focus our analysis on, we explore the community structures for Markov time $\in [0.8, 1.15]$ (SI Fig. S13). As Markov time increases, the major change for all three networks is the merging of the blue and orange modules into a single blue one, which happens at different Markov times for each network. These modules correlate with the metadata. The merged blue module contains the lawyers in the Boston and Providence offices, who split into corporate lawyers (blue) and litigators (orange) when we look for finer communities. We tabulate this for G_{c} in Table S4. Our focus rests mainly on the novelty of the pink and red modules of G_{c}, which (1) persist across Markov times and (2) have no equivalent in G_{fo}. Thus, the choice of Markov time does not qualitatively affect our analysis. We pick 0.9 since the finer communities provide insight into how individuals are grouped by work roles, and how friendship groups bridge these divisions.

Supplementary Note S3.3 The communities of G_{c}

To understand the origins of the community structure of G_{c}, we compare it to that of the co-work and friendship networks G_{w} and G_{f}. We find communities corresponding to those of G_{c} in both networks, albeit for different Markov times (SI Fig. S14).

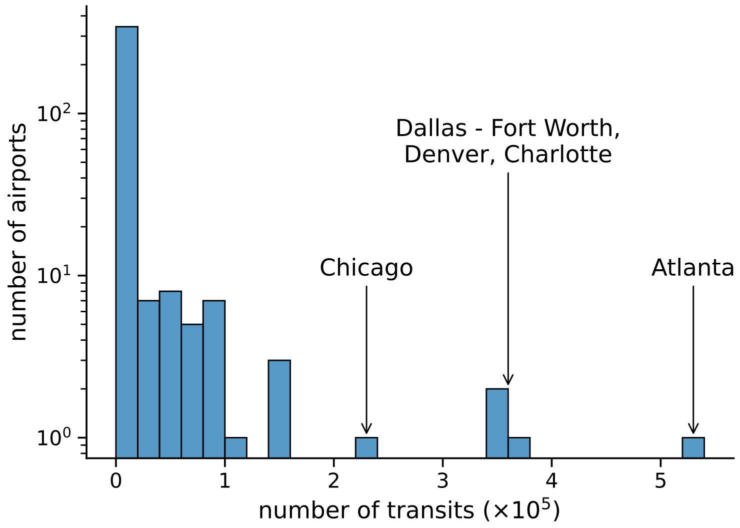

Figure S1: **Distribution of transit volume.** We highlight the 5 largest airports by transit volume.

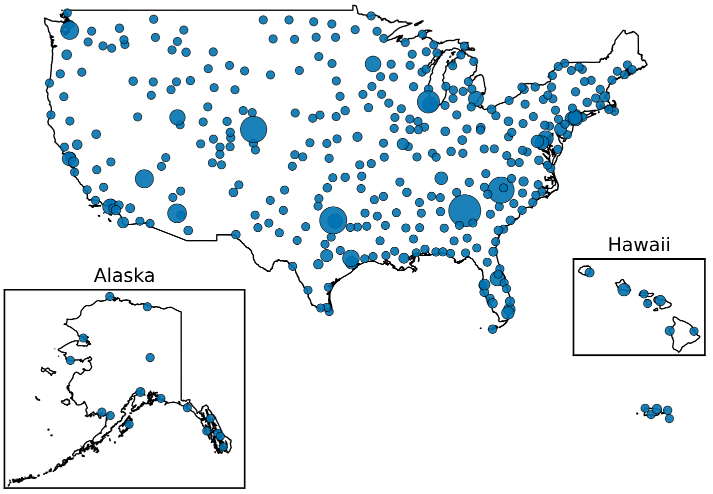

Figure S2: **Map of airports.** A map of all the airports with at least one transit through them. The size of the marker indicates the number of transits. For a compact map, we place Alaska and Hawaii as insets and do not plot the Virgin Islands, Guam, American Samoa, and Commonwealth of the Northern Mariana Islands.

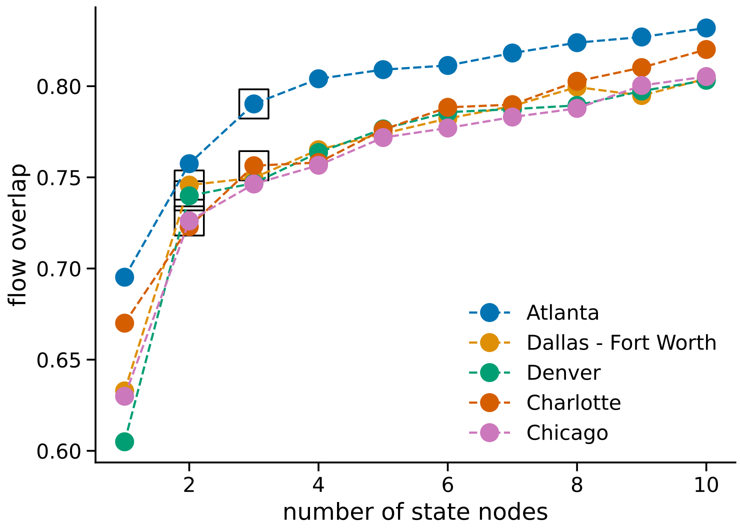

Figure S3: **Creating state nodes for large airports.** Flow overlap (y-axis) as a function of the number of state nodes (x-axis) for the five largest airports.

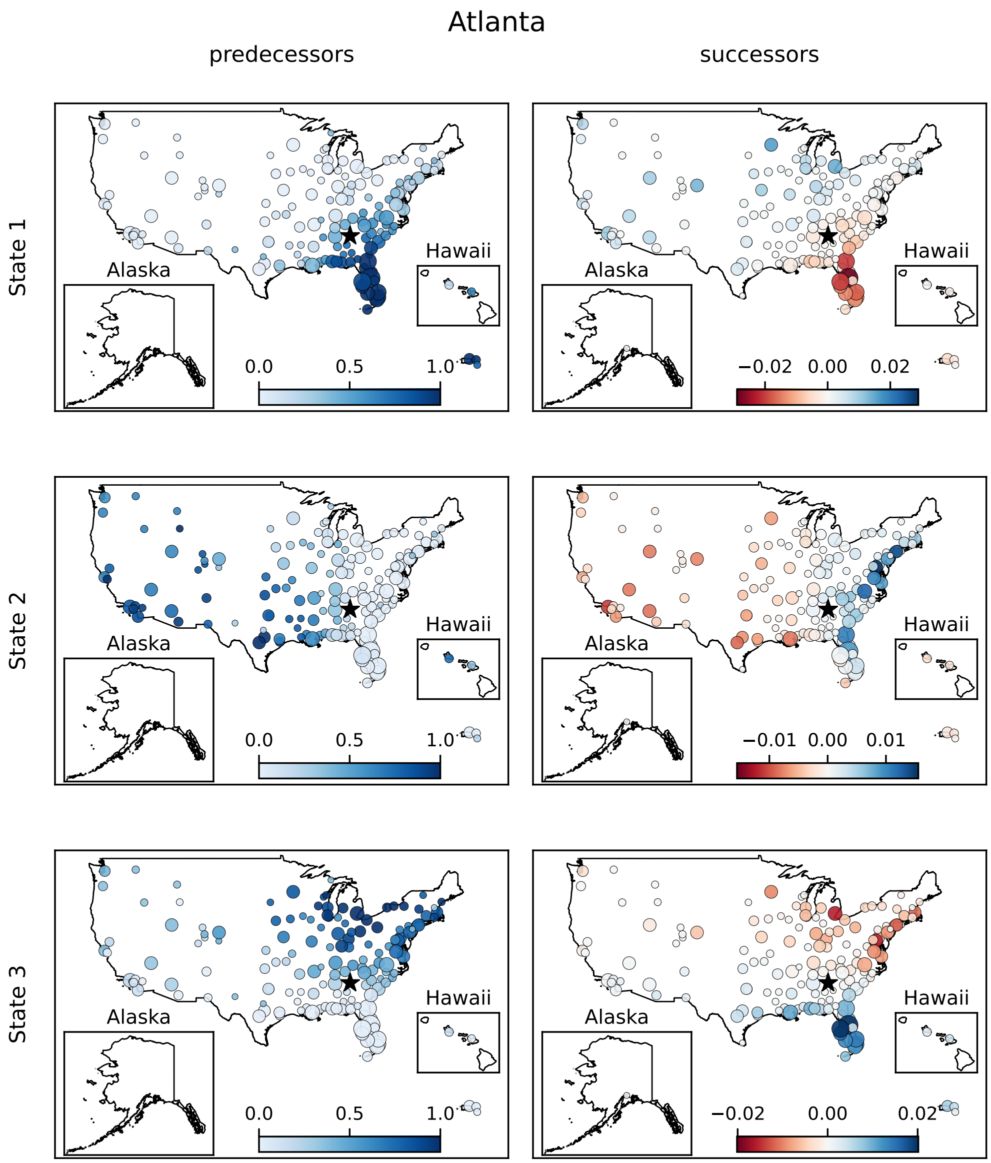

Figure S4: **State nodes of Atlanta.** Predecessors and successors are sized by their traffic with the first-order node and colored by their importance to the state node. We explain this in detail in the text.

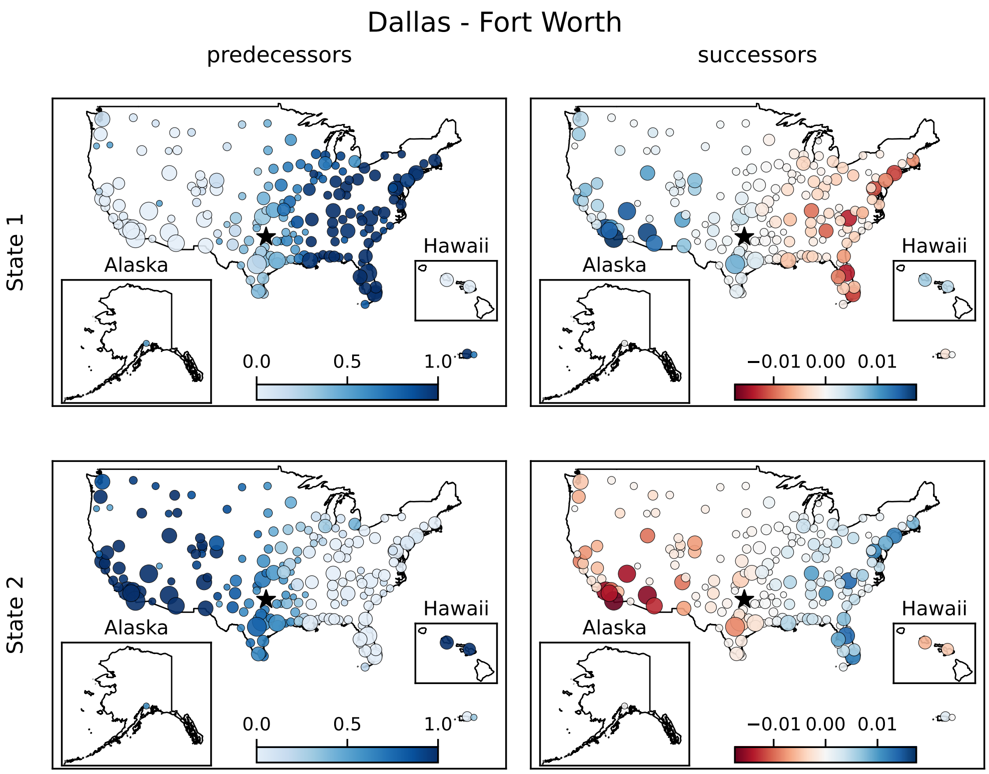

Figure S5: **State nodes of Dallas - Fort Worth.** The interpretation of marker size and color is the same as Fig.S4.

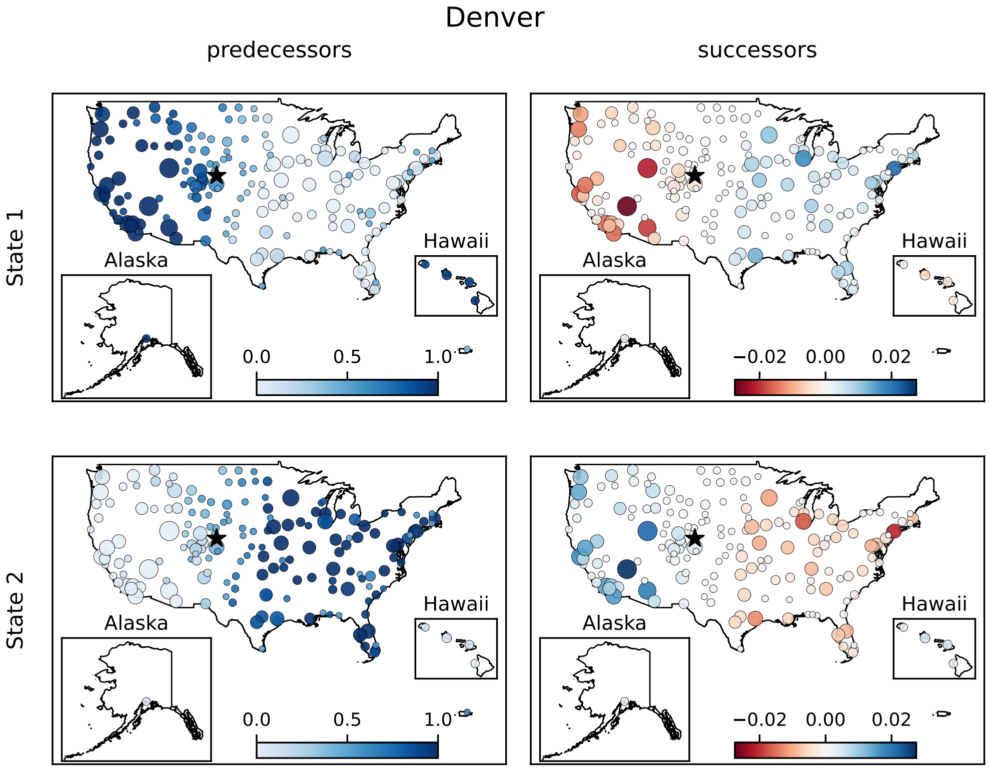

Figure S6: **State nodes of Denver.** The interpretation of marker size and color is the same as Fig.S4.

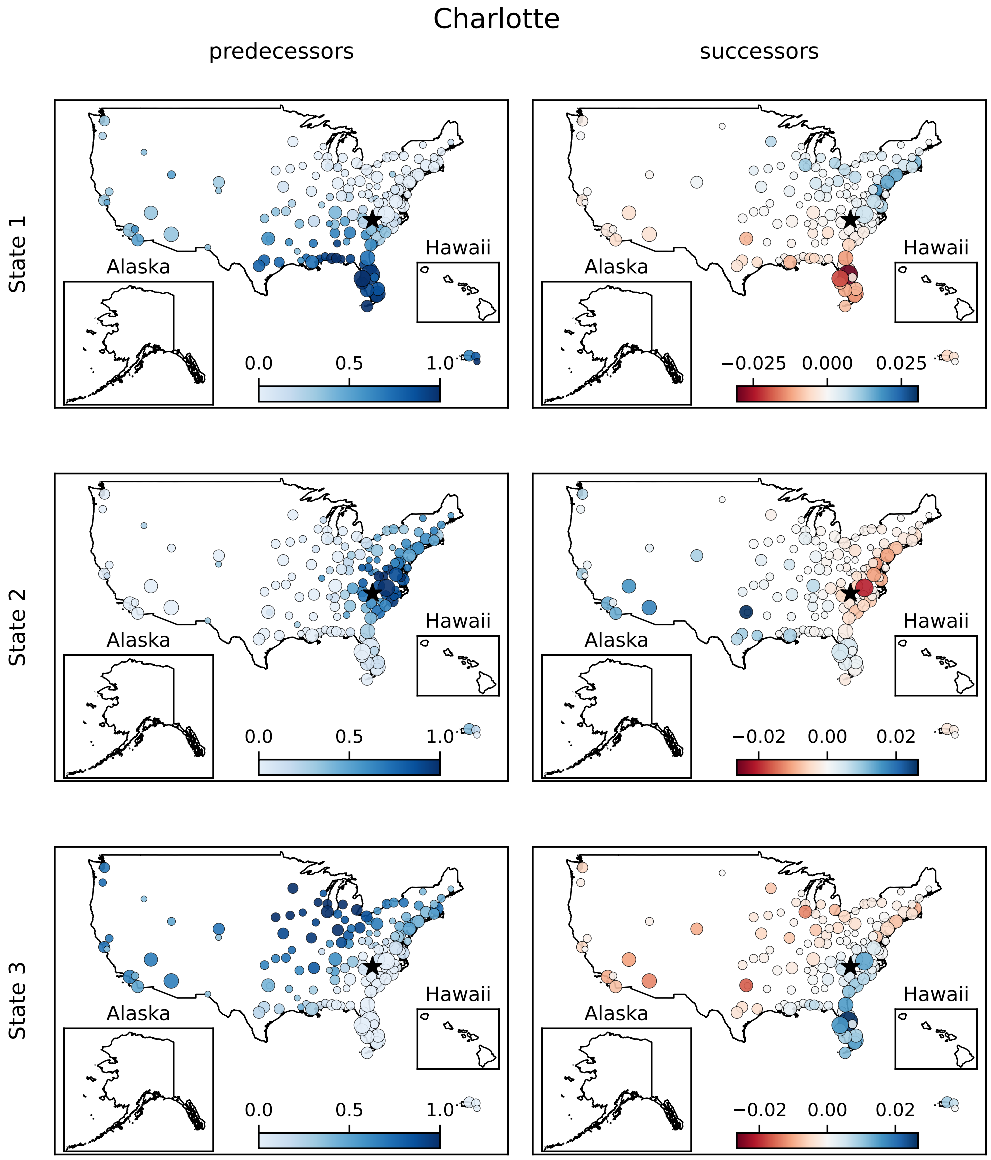

Figure S7: **State nodes of Charlotte.** The interpretation of marker size and color is the same as Fig.S4.

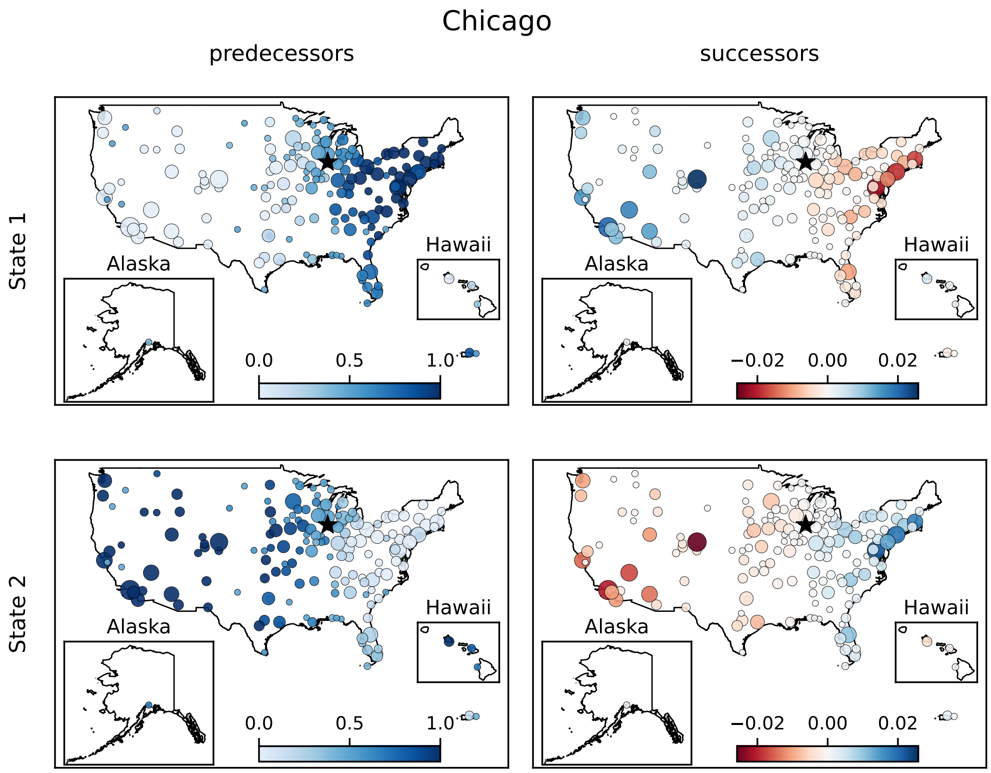

Figure S8: **State nodes of Chicago.** The interpretation of marker size and color is the same as Fig.S4.

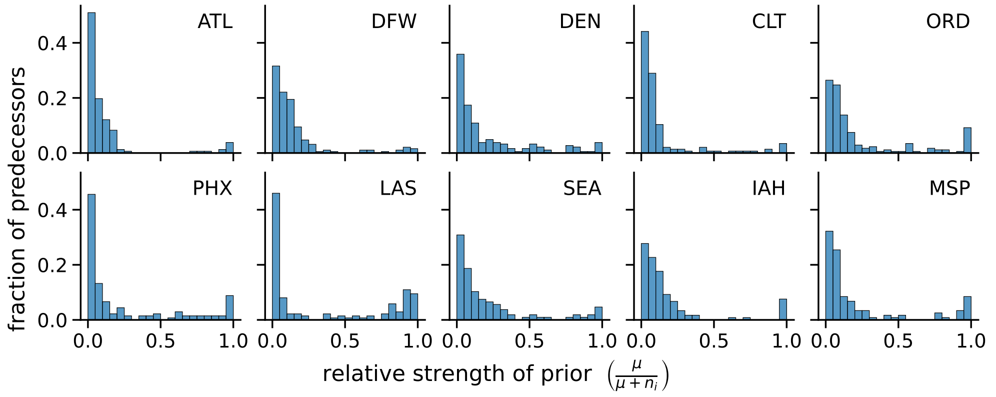

Figure S9: **Distribution of relative strength of prior**

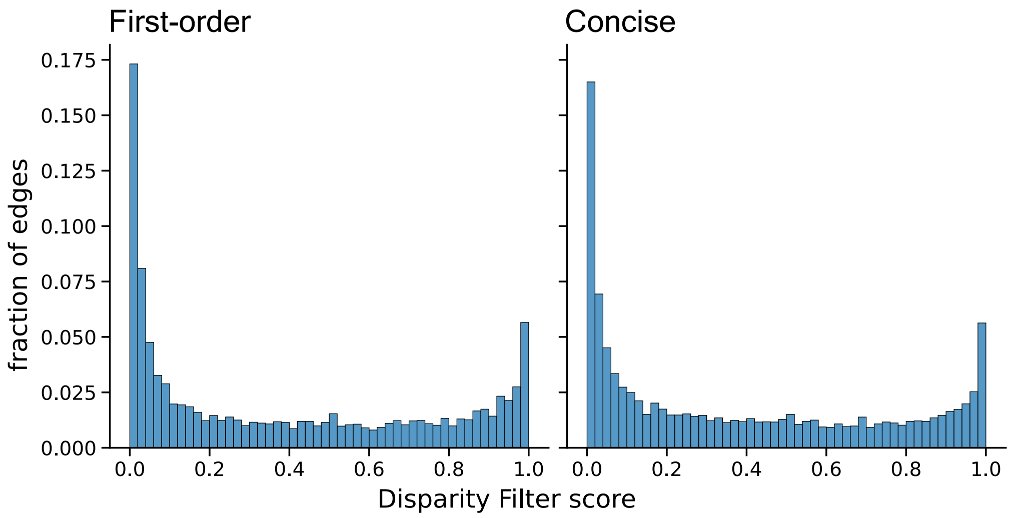

Figure S10: **Distribution of DF score.** Distribution of the Disparity Filter score of the edges in the first-order (left) and concise (right) networks.

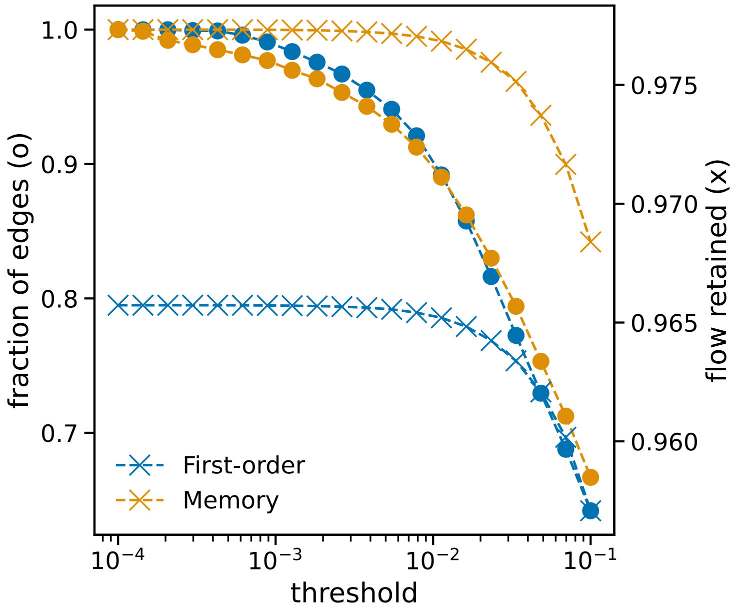

Figure S11: **Sweeping DF score threshold** The fraction of edges (left x-axis, circle markers) and flow (right x-axis, cross markers) retained as a function of threshold for the first-order (blue) and concise (orange) networks.

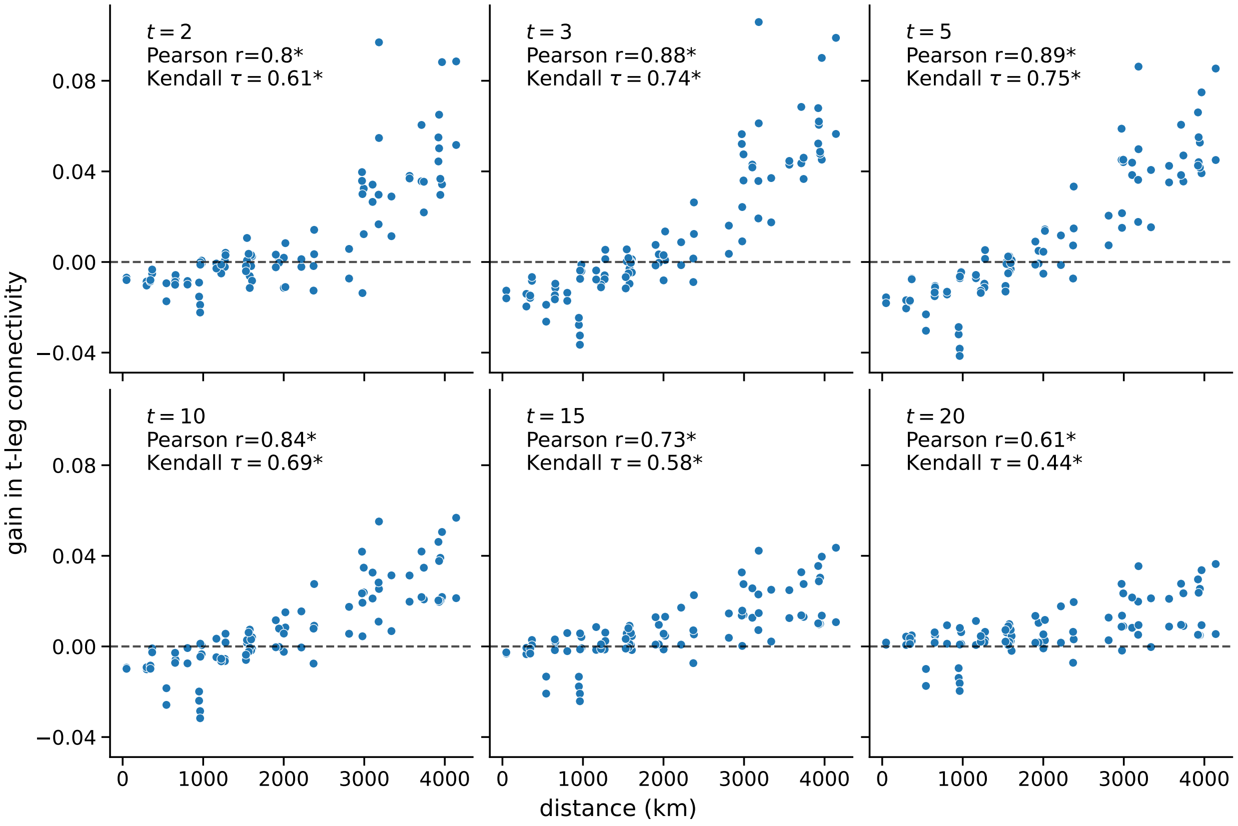

Figure S12: **Gain in connectivity.** The gain in t-leg connectivity (y-axis) for $t \in [2, 20]$ against the distance between o and d (x-axis) for (o, d) pairs of airports ranked 11-20 by number of transits. The black dashed line denotes no gain. * : p-value$< 10^{-8}$.

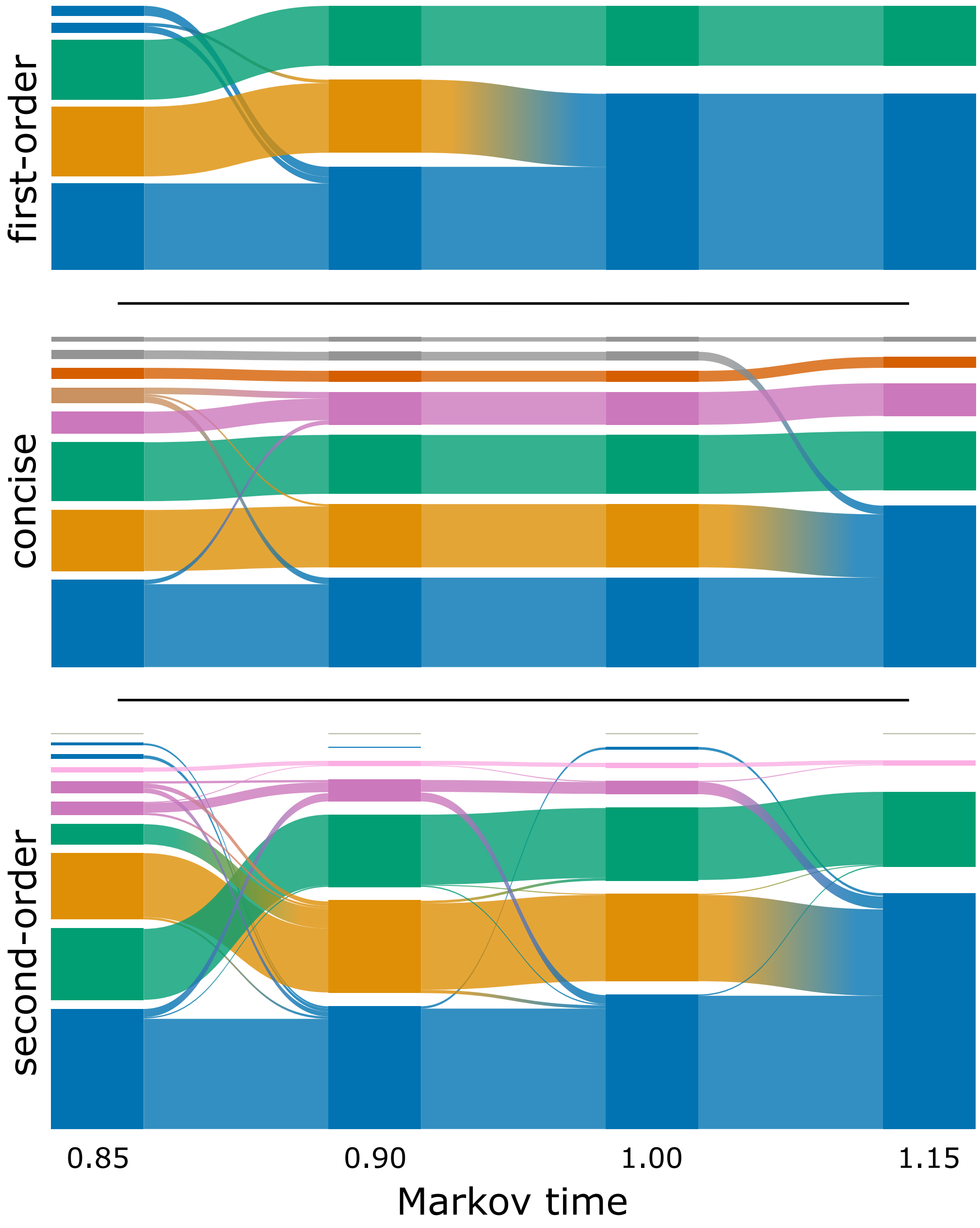

Figure S13: **Community structures across Markov times.** The changes in the community structure of G_{fo} (top), G_{c} (middle) and G_{so} (bottom) networks for Markov time $\in [0.85, 1.15]$. Each block represents a module, with the colors capturing similarity. The width of the blocks and flow lines indicate the number of (state) nodes. The figures are generated using the tool at `https://www.mapequation.org/alluvial/`.

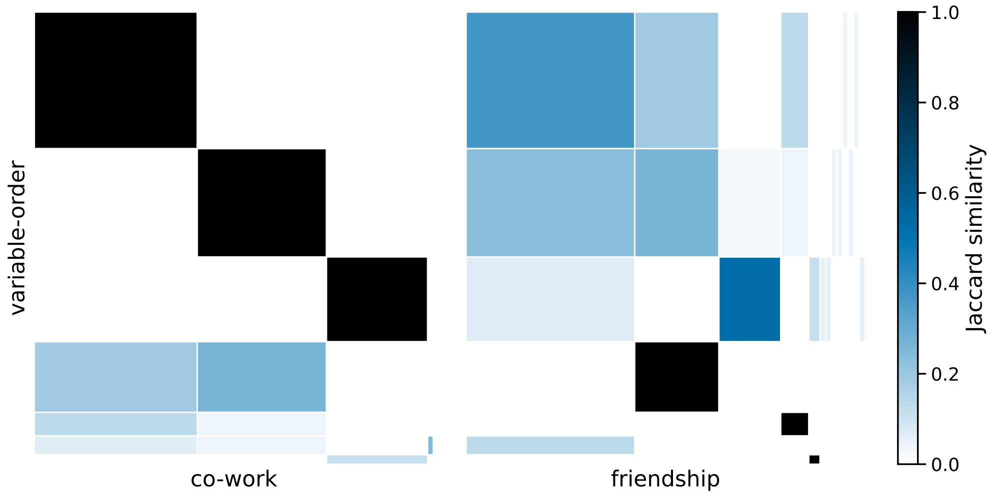

Figure S14: **Origins of the communities of $G_{\mathbf{c}}$** Jaccard similarity the communities of G_{c} (y-axis) with those of G_{w} at Markov time 1 (x-axis, left) and G_{f} at Markov time 1.2 a (x-axis, right). The height (resp. width) of each cell is proportional to the size of the community in G_{c} (resp. G_{w} and G_{f}).

Table S1: **Largest transit hubs.** The 10 largest airports with number of state nodes and flow overlap in G_c.

IATA code	Airport	# transits	# state nodes	flow overlap
ATL	Hartsfield-Jackson Atlanta	529,581	2	0.76
DFW	Dallas/Fort Worth	369,169	2	0.75
DEN	Denver	345,731	2	0.74
CLT	Charlotte Douglas	343,534	2	0.72
ORD	Chicago O'Hare	235,590	2	0.73
PHX	Phoenix Sky Harbor	159,261	5	0.70
LAS	Harry Reid	145,290	5	0.70
SEA	Seattle/Tacoma	140,914	2	0.72
IAH	George Bush Intercontinental	116,158	2	0.72
MSP	Minneapolis-St Paul	98,626	3	0.73

Table S2: **Airports ranked 11-20 by transit volume**

IATA code	Airport	# transits
SLC	Salt Lake City	93,889
LAX	Los Angeles	93,849
BWI	Baltimore/Washington	90,492
DTW	Detroit Metro Wayne County	88,438
MDW	Chicago Midway	87,508
DAL	Dallas Love Field	80,362
MCO	Orlando	78,957
LGA	LaGuardia	67,595
DCA	Ronald Reagan Washington	66,595
SFO	San Francisco	64,417

Table S3: **Metadata in the Lazega dataset.** Cross-tabulation of office location (rows) and practice (columns) of the 71 lawyers in the data.

	Corporate	Litigation	Total
Boston	19	29	48
Hartford	8	11	19
Providence	3	1	4
Total	30	41	71

Table S4: **Community structure and metadata in $G_{\mathbf{fo}}$.** Cross-tabulation of the module (rows) and metadata on office location and practice (columns) of the nodes in the first-order network. Corp. and lit. stand for corporate and litigation respectively.

	Boston		Hartford		Providence		
Module	Corp.	Lit.	Corp.	Lit.	Corp.	Lit.	Total
1	1	29	0	0	0	1	31
2	18	0	1	0	3	0	22
3	0	0	7	11	0	0	18
Total	19	29	8	11	3	1	71

REFERENCES AND NOTES

1. M. Newman, *Networks* (Oxford Univ. Press, 2018).

2. R. Lambiotte, M. T. Schaub, *Modularity and Dynamics on Complex Networks* (Cambridge Univ. Press, 2021).

3. F. Chierichetti, R. Kumar, P. Raghavan, T. Sarlos, "Are web users really markovian?," in *Proceedings of the 21st International Conference on World Wide Web* (Association for Computing Machinery, 2012), pp. 609–618.

4. P. Kareiva, N. Shigesada, Analyzing insect movement as a correlated random walk. *Oecologia* **56**, 234–238 (1983).

5. M. R. Meiss, F. Menczer, S. Fortunato, A. Flammini, A. Vespignani, "Ranking web sites with real user traffic," in *Proceedings of the 2008 International Conference on Web Search and Data Mining* (Association for Computing Machinery, 2008), pp. 65–76.

6. R. West, J. Leskovec, "Human wayfinding in information networks," in *Proceedings of the 21st International Conference on World Wide Web* (Association for Computing Machinery, 2012), pp. 619–628.

7. C. T. Butts, Revisiting the foundations of network analysis. *Science* **325**, 414–416 (2009).

8. M. Rosvall, A. V. Esquivel, A. Lancichinetti, J. D. West, R. Lambiotte, Memory in network flows and its effects on spreading dynamics and community detection. *Nat. Commun.* **5**, 4630 (2014).

9. J. Xu, T. L. Wickramarathne, N. V. Chawla, Representing higher-order dependencies in networks. *Sci. Adv.* **2**, e1600028 (2016).

10. I. Scholtes, "When is a network a network? Multi-order graphical model selection in pathways and temporal networks," in *Proceedings of the 23rd ACM SIGKDD International Conference on Knowledge Discovery and Data Mining* (Association for Computing Machinery, 2017), pp. 1037–1046.

11. R. Lambiotte, M. Rosvall, I. Scholtes, From networks to optimal higher-order models of complex systems. *Nat. Phys.* **15**, 313–320 (2019).

12. C. Gote, G. Casiraghi, F. Schweitzer, I. Scholtes, Predicting variable-length paths in networked systems using multi-order generative models. *Appl. Netw. Sci.* **8**, 68 (2023).

13. M. Saebi, J. Xu, L. M. Kaplan, B. Ribeiro, N. V. Chawla, Efficient modeling of higher-order dependencies in networks: From algorithm to application for anomaly detection. *EPJ Data Sci.* **9**, 15 (2020).

14. J. Queiros, C. Coquidé, F. Queyroi, Toward random walk-based clustering of variable-order networks. *Netw. Sci.* **10**, 381–399 (2022).

15. C. H. Q. Ding, T. Li, M. I. Jordan, Convex and semi-nonnegative matrix factorizations. *IEEE Trans. Pattern Anal. Mach. Intell.* **32**, 45–55 (2008).

16. V. Salnikov, M. T. Schaub, R. Lambiotte, Using higher-order Markov models to reveal flow-based communities in networks. *Sci. Rep.* **6**, 23194 (2016).

17. T. LaRock, V. Nanumyan, I. Scholtes, G. Casiraghi, T. Eliassi-Rad, F. Schweitzer, “Hypa: Efficient detection of path anomalies in time series data on networks,” in *Proceedings of the 2020 SIAM International Conference on Data Mining* (SIAM, 2020), pp. 460–468.

18. E. Lazega, *The Collegial Phenomenon: The Social Mechanisms of Cooperation Among Peers in a Corporate Law Partnership* (Oxford Univ. Press, 2001).

19. D. Edler, A. Holmgren, M. Rosvall, The MapEquation software package (2024); https://mapequation.org.

20. D. Edler, L. Bohlin, M. Rosvall, Mapping higher-order network flows in memory and multilayer networks with infomap. *Algorithms* **10**, 112 (2017).

21. C. Zhai, J. Lafferty, C. Zhai, J. Lafferty, A study of smoothing methods for language models applied to information retrieval. *ACM Trans. Inf. Syst.* **22**, 179–214 (2004).

22. U.S. Bureau of Transportation Statistics, Airline Origin and Destination Survey (2023); https://transtats.bts.gov/DataIndex.asp [accessed 13 June 2024].

23. M. Á. Serrano, M. Boguná, A. Vespignani, Extracting the multiscale backbone of complex weighted networks. *Proc. Natl. Acad. Sci. U.S.A.* **106**, 6483–6488 (2009).

24. S. Fortunato, Community detection in graphs. *Phys. Rep.* **486**, 75–174 (2010).

25. C. R. Harris, K. J. Millman, S. J. van der Walt, R. Gommers, P. Virtanen, D. Cournapeau, E. Wieser, J. Taylor, S. Berg, N. J. Smith, R. Kern, M. Picus, S. Hoyer, M. H. van Kerkwijk, M. Brett, A. Haldane, J. F. del Río, M. Wiebe, P. Peterson, P. Gérard-Marchant, K. Sheppard, T. Reddy, W. Weckesser, H. Abbasi, C. Gohlke, T. E. Oliphant, Array programming with NumPy. *Nature* **585**, 357–362 (2020).

26. W. McKinney, "Data Structures for Statistical Computing in Python," in *Proceedings of the 9th Python in Science Conference*, S. van der Walt, J. Millman, Eds. (SciPy, 2010), pp. 51–56.

27. A. A. Hagberg, D. A. Schult, P. J. Swart, "Exploring Network Structure, Dynamics, and Function using NetworkX," in *Proceedings of the 7th Python in Science Conference*, G. Varoquaux, T. Vaught, J. Millman, Eds. (SciPy, 2008), pp. 11–15.

28. P. Virtanen, R. Gommers, T. E. Oliphant, M. Haberland, T. Reddy, D. Cournapeau, E. Burovski, P. Peterson, W. Weckesser, J. Bright, S. J. van der Walt, M. Brett, J. Wilson, K. J. Millman, N. Mayorov, A. R. J. Nelson, E. Jones, R. Kern, E. Larson, C J Carey, İ. Polat, Y. Feng, E. W. Moore, J. V. Plas, D. Laxalde, J. Perktold, R. Cimrman, I. Henriksen, E A Quintero, C. R. Harris, A. M. Archibald, A. H. Ribeiro, F. Pedregosa, P. van Mulbregt, SciPy 1.0 Contributors, SciPy 1.0: Fundamental algorithms for scientific computing in Python. *Nat. Methods* **17**, 261–272 (2020).

29. M. de Domenico, Multilayer network repository, https://manliodedomenico.com/data.php [accessed 21 June 2024].

30. M. Coscia, Network backboning (2017); https://michelecoscia.com/?page_id=287.

31. C. Ding, X. He, H. D. Simon, "On the equivalence of nonnegative matrix factorization and spectral clustering," in *Proceedings of the 2005 SIAM International Conference on Data Mining* (SIAM, 2005), pp. 606–610.

32. M. Rosvall, C. T. Bergstrom, Maps of random walks on complex networks reveal community structure. *Proc. Natl. Acad. Sci. U.S.A.* **105**, 1118–1123 (2008).

33. M. Kheirkhahzadeh, A. Lancichinetti, M. Rosvall, Efficient community detection of network flows for varying Markov times and bipartite networks. *Phys. Rev. E* **93**, 032309 (2016).
